# ChatGPT: Friend or foe in medical writing? An example of how ChatGPT can be utilized in writing case reports

**DOI:** 10.1016/j.sipas.2023.100185

**Published:** 2023-06-02

**Authors:** Wai Lone Jonathan Ho, Bilal Koussayer, Joseph Sujka

**Affiliations:** a USF Health Morsani College of Medicine, 560 Channelside Dr, Tampa, FL 33602, United States; b USF Department of General Surgery 2 Tampa General Circle, 7th Floor Tampa, FL 33606, United States

**Keywords:** ChatGPT, Artificial intelligence, Surgical education, Case report, Research, Hernia repair

## Abstract

ChatGPT is a chatbot built on a natural language processing model which can generate human-like responses to prompts given to it. Despite its lack of domain-specific training, ChatGPT has developed remarkable accuracy in interpreting clinical information. In this article, we aim to assess what role ChatGPT can serve in medical writing. We recruited a first-year medical student with no prior experience in writing case reports to write a case report on a complex surgery with the assistance of ChatGPT. After a thorough evaluation of its responses, we believe that ChatGPT is a powerful medical writing tool that can be used to generate summaries, proofread, and provide valuable medical insight. However, ChatGPT is not a substitute for a study author due to several significant limitations, and should instead be used in conjunction with the author during the writing process. As the impact of natural language processing models such as ChatGPT grows, we suggest that guidelines be established on how to better utilize this technology to improve clinical research rather than outright prohibiting its usage.

## Highlights

• ChatGPT can proofread and summarize medical reports without omitting key info.
• The interactive nature of ChatGPT can lead to new medical insights.
• ChatGPT can provide contextualized answers based on the conversation history.
• ChatGPT provides false citations that cannot be located in any medical database.
• There is a lack of clear guidelines regarding the usage of ChatGPT in research.

## Introduction

ChatGPT is an OpenAI-developed chatbot built on an advanced natural language processing (NLP) model designed to generate human-like responses to prompts [1]. In the field of medicine, its achievements have been impressive, with it being capable of passing medical examinations, diagnosing, and proposing treatments for complex conditions [1], [2], [3]. Furthermore, it has been used to produce text that is indistinguishable from human authors by experts in the field [3]. Its use has been met with mixed reactions, with many becoming concerned that it can be utilized irresponsibly to plagiarize or “shortcut” tasks such as writing. Meanwhile, others have advocated that ChatGPT has a role to play in medicine and writing, with it being listed as an author in several preprints at the time of writing [4]. Case reports often serve to highlight educational cases for a wide audience and provide a valuable opportunity to contribute new ideas to medicine. Therefore, we wanted to test ChatGPT's capabilities in assisting a medical student in writing a case report and assess the benefits and drawbacks of such a practice. We also evaluate methods to improve the accuracy of ChatGPT's responses and provide suggestions for more efficient utilization of ChatGPT.

## Materials and methods

In this study, a first-year medical student with no prior experience in writing case reports was recruited to write a case report using ChatGPT, and a patient who underwent robotic hernia repair due to a slipped Nissen fundoplication was used as the case study. Patient information was deidentified and any date and location identifiers were removed.

**Fig. 1:**
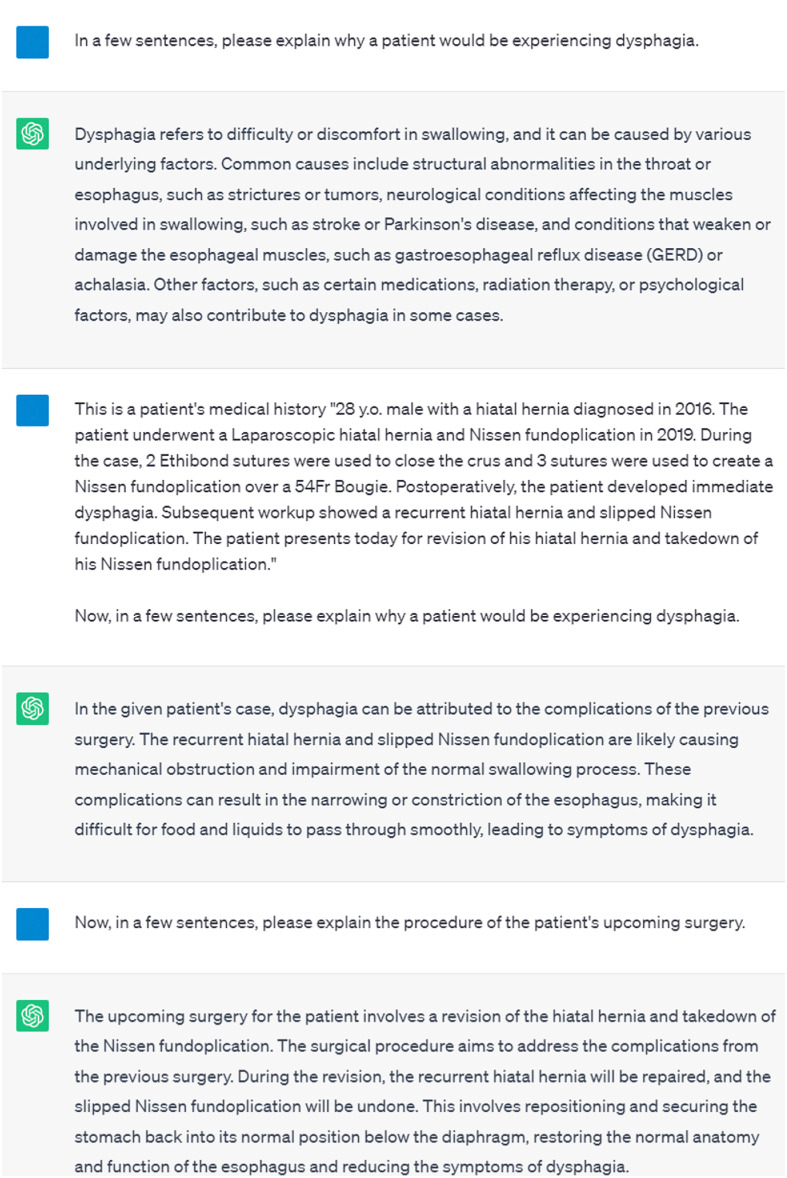
Incorporating a Patient's Medical History in the Prompt.

We first conducted a simple two-step experiment to investigate the importance of priming ChatGPT with relevant patient information. In the initial step, we evaluated ChatGPT's response to a medical question under two conditions, one in which the patient's medical history was omitted in the prompt, and another in which it was included (Fig. 1). We then posed a follow-up question to gage its ability to maintain an understanding of the patient's case beyond the initial prompt.

When writing the case report, the student incorporated a similar approach to prime ChatGPT by first providing the patient's medical history and operation note as raw input (Fig. A.2, A.3, and A.4). ChatGPT was then prompted to proofread and summarize the raw inputs, which, while not necessary for priming ChatGPT, effectively reduced the time required for manual proofreading and editing.

Moreover, the student was careful in providing prompts with the appropriate wording to elicit the most accurate responses from ChatGPT. With reference to our previous example, ChatGPT was requested to “proofread” and “summarize" the operation note to avoid losing key information and to ensure that the correct medical terminology was maintained.

**Fig. A.1:**
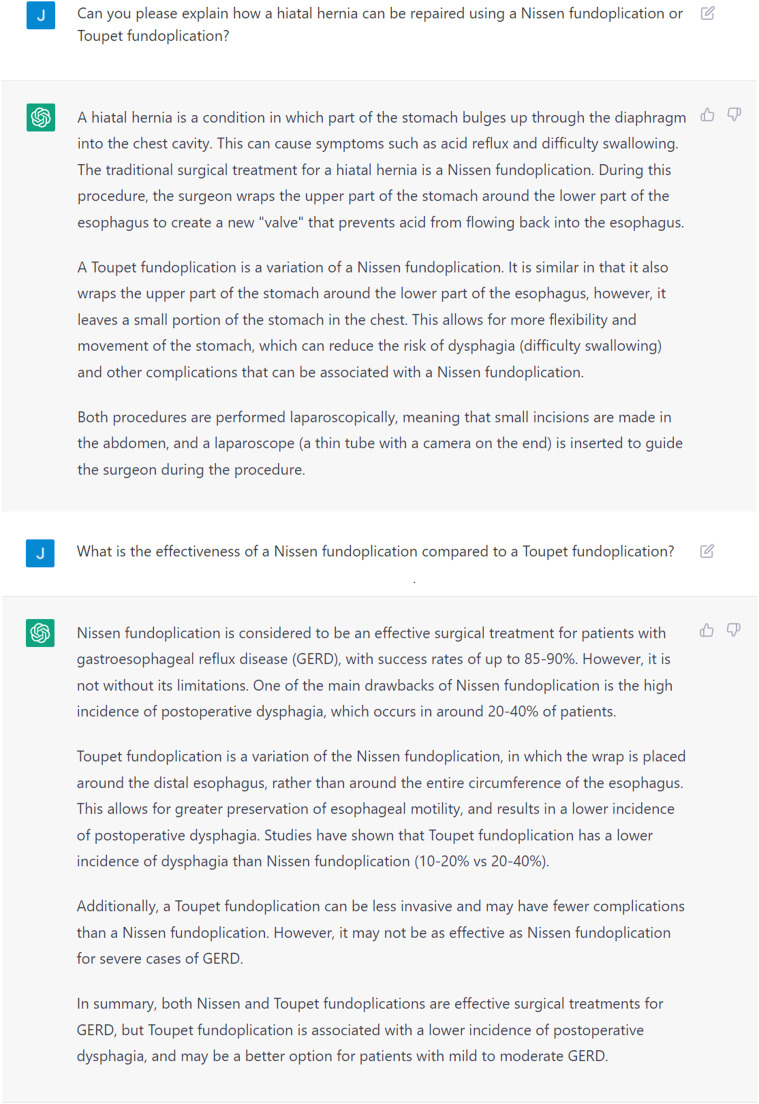
Responses Generated by ChatGPT for the Introduction.

**Fig. A.2:**
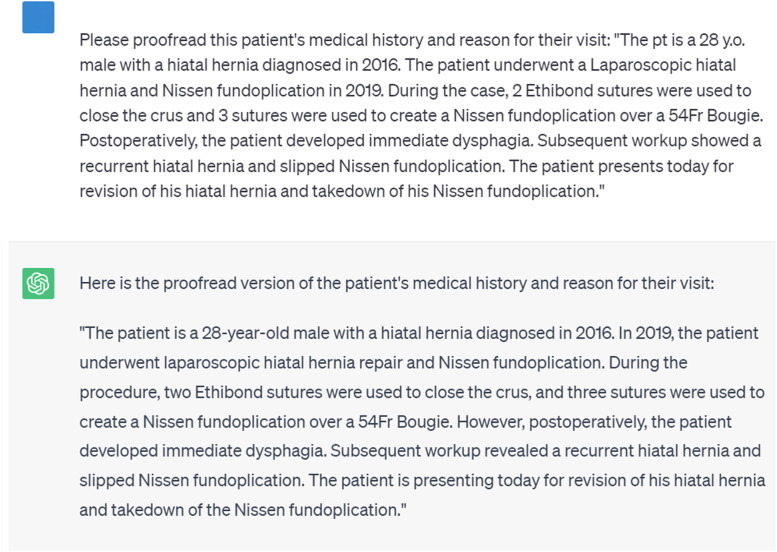
Response Generated by ChatGPT for the Case Presentation.

Subsequently, ChatGPT was asked questions relevant to the case, and its responses were adapted to write the introduction, discussion, and conclusion sections (Fig. A.1, A.5, and A.6). References were found on online databases and cited.

The responses from ChatGPT that were used for the case report are italicized, and manual human edits are in standard text. The full case report can be found in Appendix A.

## Results

### Evaluating ChatGPT's performance with contextual information

After incorporating the patient's medical history, ChatGPT utilized aspects of the case to explain how mechanical obstruction caused by the slippage of the Nissen fundoplication led to symptoms of dysphagia, omitting less likely causes such as “neurological conditions” and “radiation therapy” that were mentioned in the first response. Furthermore, in a follow-up question, ChatGPT demonstrated its ability to maintain an understanding of the patient's planned surgery by explaining the complex procedure of repairing a hiatal hernia and slipped Nissen fundoplication.

### Generating the discussion using responses from ChatGPT

*The treatment for a slipped Nissen fundoplication can vary depending on the severity of the symptoms and the underlying* mechanism of failure [5]. *Repositioning a slipped Nissen fundoplication can be ineffective as the wrap may slip again over time and it may not correct the underlying problems that led to the patient's symptoms, such as acid reflux or dysphagia* [6], [7], [8]*. Therefore, it is often recommended to convert a slipped Nissen fundoplication to a Toupet fundoplication, which involves a partial wrap of the stomach around the esophagus,* that has been associated with lower rates of reoperation and dysphagia [9,10]. The long-term outcomes of converting a Nissen fundoplication have not been well described in the literature and more research is needed to better determine superior management techniques.

**Fig. 2:**
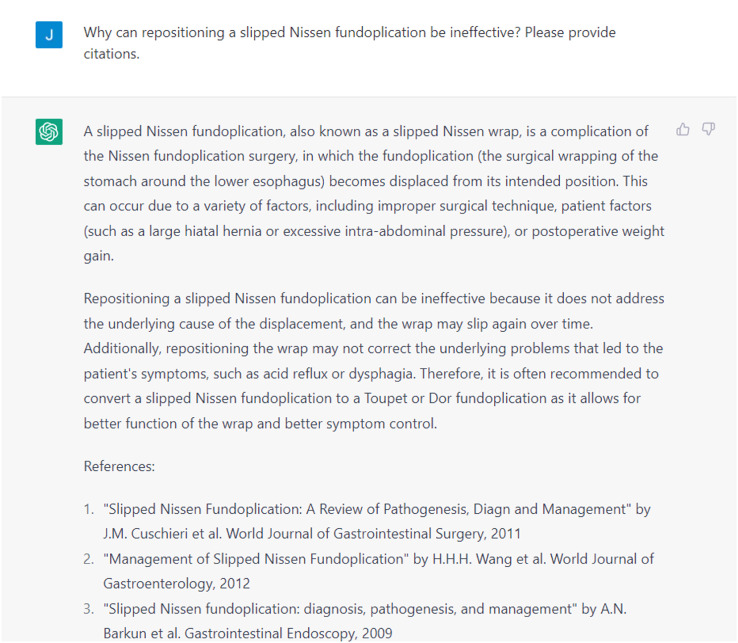
Example of a Response Generated by ChatGPT for the Discussion.

## Discussion

Previous studies have shown that engaging in ‘dialogue’ with ChatGPT enhances its performance by conveying user intent and providing relevant contextual information [11,12]. Our two-step experiment (Fig. 1) and written case report (Appendix A) reinforce these findings and demonstrate ChatGPT's improved performance in answering questions specific to the patient's case. Therefore, we recommend incorporating a similar approach when drafting a case report by: (1) priming ChatGPT with key patient information, and (2) asking ChatGPT a series of related questions within the same conversation to maintain contextual coherence.

Through this interactive dialog, the medical student found ChatGPT to be a helpful tool for simplifying complex medical information and learning unfamiliar topics, thus enhancing the learning process. For instance, when the student sought clarification on why a slipped Nissen fundoplication required conversion to a Toupet fundoplication, ChatGPT was able to explain why repositioning a slipped Nissen fundoplication would not resolve this patient's specific symptoms of “acid reflux or dysphagia” (Fig. 2). This valuable background information guided the student towards a more focused literature review using medical databases. Importantly, the student took care to critically evaluate ChatGPT's responses and ensure that their literature review was not overly narrow in scope. As many of ChatGPT's responses seem clinically plausible at first glance, it is easy to overlook potential inaccuracies. Notably, most of ChatGPT's responses were supported by credible studies from online medical databases, which were also cited in the case report.

**Fig. A.3:**
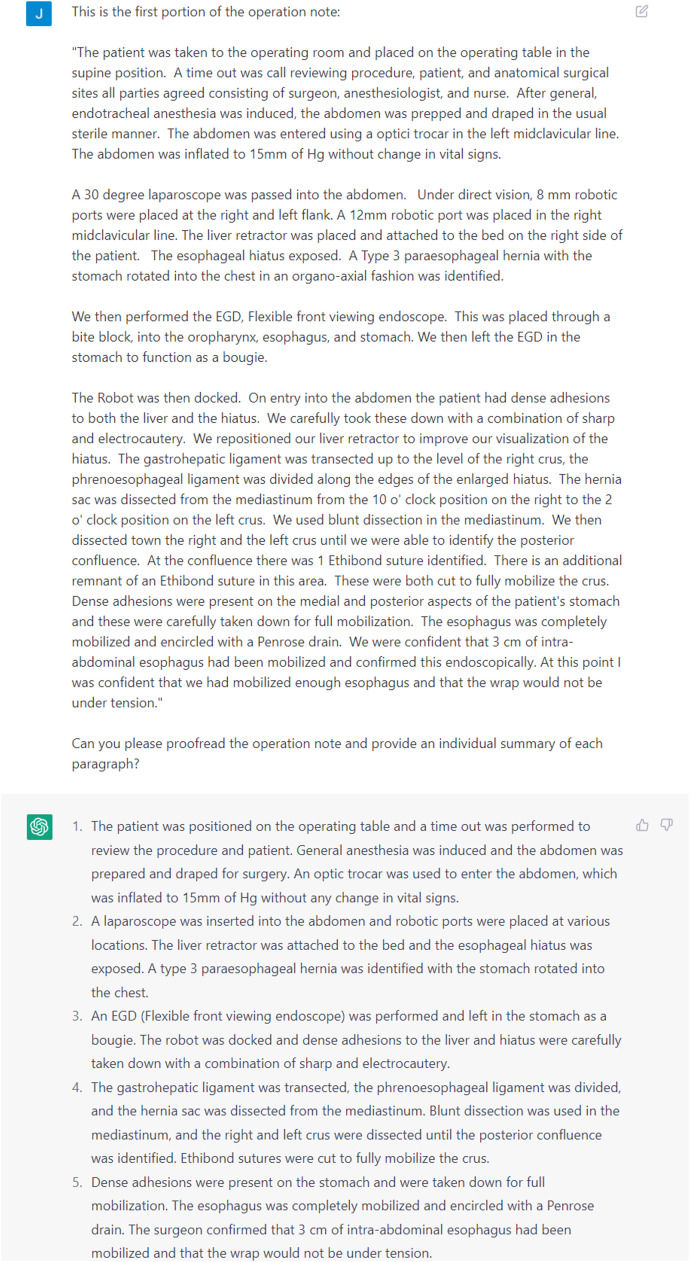
Response Generated by ChatGPT for the Case Presentation.

While large language models such as ChatGPT have been successfully utilized to summarize mediums such as news articles and online posts [13,14], we have shown that it can also be used to summarize complex and unorganized medical reports without losing important information (Fig. A.3). Its ability to proofread large volumes of text while maintaining accurate medical terminology significantly reduces the effort required to write a case report. According to the medical student's estimation, drafting the case presentation section took two hours, while an additional four hours were spent conducting a focused literature review and drafting the remaining sections. Based on our research group's experience, this case report was completed more efficiently than the average fifteen hours of work typically required by a medical student.

Despite its benefits, we have also identified several significant limitations, the most glaring being ChatGPT's inability to provide credible citations. When asked to provide citations, ChatGPT “hallucinates” false citations that seem convincingly real (Fig. 2) but cannot be located in any medical database [1]. “Hallucination” by ChatGPT has been extensively characterized and occurs because ChatGPT retrieves information from multiple sources it has been trained on, rather than relying on a single source [15], [16], [17]. As the accuracy of its responses depends on the validity of its training dataset, a dataset containing flawed or outdated information can result in an inaccurate model that provides factually incorrect answers [18]. OpenAI has acknowledged these limitations, openly stating that ChatGPT is trained on a dataset that was collected in 2020 and that it exhibits several racial, gender, and religious biases [15,19]. Therefore, users must be diligent in ensuring that the responses generated by ChatGPT are crosschecked with actual studies. However, we should also acknowledge the ongoing progress being made by OpenAI to address its shortcomings, with substantial improvements seen in GPT-4 compared to GPT-3.5 [20].

Additionally, there are also privacy concerns as the data collected by ChatGPT may be stored [21]. As authors of case reports often work with patient information, care must be taken to deidentify patient data.

Another challenge to utilizing ChatGPT in medical writing is the lack of rules and guidelines available to follow. Some feel that its usage should be encouraged while others have gone so far as to consider the use of ChatGPT to be a form of plagiarism due to the lack of transparency behind its sources [4]. Many journals have not yet come to a consensus on what role ChatGPT will play in publishing research.

## Conclusion

ChatGPT is a powerful medical writing tool that can be used to generate summaries, proofread, and provide valuable medical insight. However, it needs to be used with caution and an understanding of its limitations, and should not be used as a substitute for a study author. Undoubtedly, the impact we are seeing of NLP models such as ChatGPT on scientific research and medicine is only going to grow. Instead of fearing its effects, we should develop frameworks and guidelines on how to better utilize this technology to improve clinical research and medicine.

## Disclosure

No funds were received in support of this work. No benefits in any form have been or will be received from any commercial party related directly or indirectly to the subject of this manuscript.

## Declaration of Competing Interest

The authors declare that they have no known competing financial interests or personal relationships that could have appeared to influence the work reported in this paper.

## Figures and Tables

**Fig. A.4:**
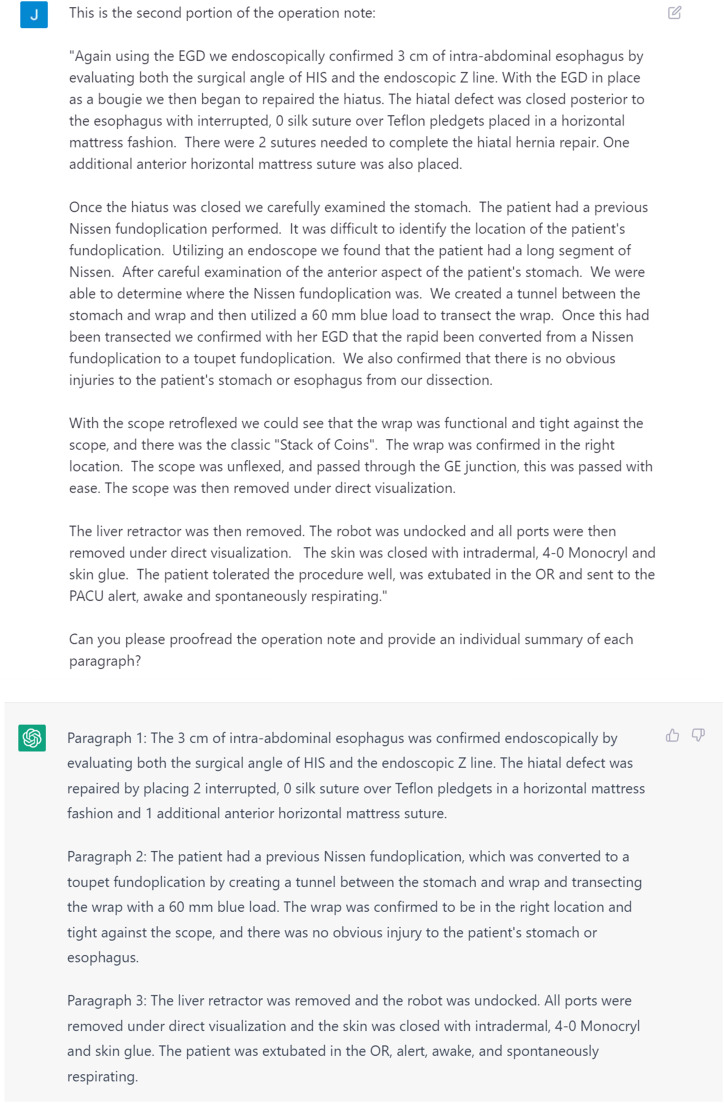
Response Generated by ChatGPT for the Case Presentation.

**Fig. A.5:**
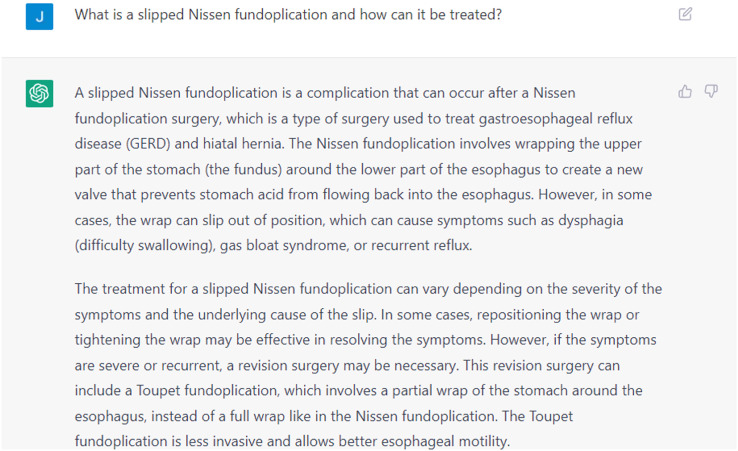
Response Generated by ChatGPT for the Discussion and Conclusion.

**Fig. A.6:**
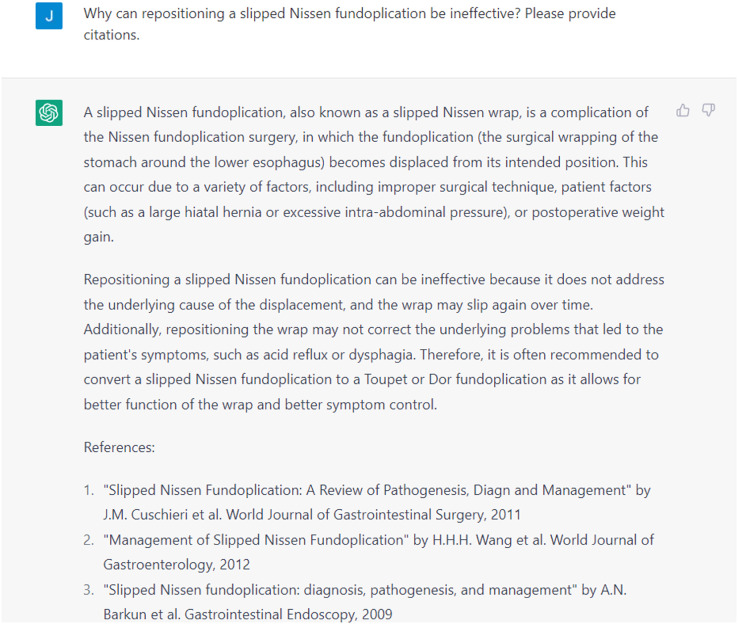
Response Generated by ChatGPT for the Discussion and Conclusion.

## Appendix

**Case Report: Robotic Hiatal hernia repair with stapled conversion from Nissen to Toupet fundoplication**

**Introduction Generated Using Responses from ChatGPT**

*Patients with hiatal hernia often experience symptoms such as refractory gastroesophageal reflux disease (GERD) and dysphagia.* The Laparoscopic Nissen Fundoplication (LNF) technique is more often and classically used to treat GERD rather than Laparoscopic Toupet Fundoplication (LTF). *However, one of the main drawbacks of LNF is the higher rates of reoperation and postoperative dysphagia after* LN*F compared to LTF* [22,23]*.* Due to possible slipping of Nissen fundoplication, conversion of a Nissen fundoplication to Toupet fundoplication is sometimes recommended in patients with recurrent hiatal hernias [5]. Here, we present a case of conversion of Nissen to Toupet fundoplication using robot surgery.

**Case Presentation Generated Using Responses from ChatGPT**

*The patient is a 28-year-old male who was diagnosed with a hiatal hernia in 2016 and underwent a laparoscopic hiatal hernia repair and Nissen fundoplication in 2019. However*, he developed dysphagia postoperatively *and subsequent workup revealed* that he had developed *a recurrent hiatal hernia and a slipped Nissen fundoplication.* He is now presenting for elective robotic surgery for a *revision of his hiatal hernia and a takedown of his Nissen fundoplication* to alleviate his symptoms of dysphagia and resolve the recurrent hiatal hernia.

After the patient was induced on general anesthesia, *a laparoscope was inserted into the abdomen and a liver retractor* was used to expose the esophageal hiatus, revealing a *Type 3 paraesophageal hernia.* An esophagogastroduodenoscopy (EGD) was performed to visualize the stomach and function as a bougie. *The patient had dense adhesions to both the liver and hiatus, which were taken down with a combination of sharp and electrocautery dissection. The gastrohepatic ligament was transected up to the level of the right crus, and the phrenoesophageal ligament was divided along the edges of the enlarged hiatus. The hernia sac was dissected from the mediastinum,* and the crus, stomach, and esophagus were completely mobilized. *The surgical team confirmed that 3* *cm of intra-abdominal esophagus had been mobilized and that the wrap would not be under tension.*

*The hiatal defect was repaired by placing two interrupted, 0 silk suture over Teflon pledgets in a horizontal mattress fashion, with one additional anterior horizontal mattress suture also placed.* Once the hiatus was closed, the stomach was carefully examined. The EGD revealed a slipped Nissen fundoplication, *which was converted to a Toupet fundoplication by creating a tunnel between the stomach and wrap and transecting the wrap with a 60* *mm blue load. The wrap was confirmed to be in the right location, with no obvious injury to the patient's stomach or esophagus.* The patient tolerated the procedure well. He subsequently followed up in the clinic with a resolution of his dysphagia and GERD without apparent complications.

**Discussion and Conclusion Generated Using Responses from ChatGPT**

*A slipped Nissen fundoplication is a postoperative complication that can occur. The Nissen fundoplication involves wrapping the fundus around the lower esophagus to create a new valve that prevents stomach acid from flowing back into the esophagus*[24]*. In some cases, the wrap can slip out of position, causing symptoms such as dysphagia, bloating, or recurrent reflux* [25,26]. *This can occur due to a variety of factors, including improper surgical technique, patient factors (such as a large hiatal hernia or excessive intra-abdominal pressure), or postoperative weight gain* [6], [7], [8]*.*

*The treatment for a slipped Nissen fundoplication can vary depending on the severity of the symptoms and the underlying* mechanism of failure [5]. *Repositioning a slipped Nissen fundoplication can be ineffective as the wrap may slip again over time and it may not correct the underlying problems that led to the patient's symptoms, such as acid reflux or dysphagia* [6], [7], [8]*. Therefore, it is often recommended to convert a slipped Nissen fundoplication to a Toupet fundoplication, which involves a partial wrap of the stomach around the esophagus,* that has been associated with lower rates of reoperation and dysphagia [9,10]. The long-term outcomes of converting a Nissen fundoplication have not been well described in the literature and more research is needed to better determine superior management techniques.
